# Local adaptation in thermal tolerance for a tropical butterfly across ecotone and rainforest habitats

**DOI:** 10.1242/bio.058619

**Published:** 2021-04-06

**Authors:** Michel A. K. Dongmo, Rachid Hanna, Thomas B. Smith, K. K. M. Fiaboe, Abraham Fomena, Timothy C. Bonebrake

**Affiliations:** 1 International Institute of Tropical Agriculture (IITA), PO Box 2008 (Messa), Yaoundé-Cameroon, Yaoundé, Cameroon; 2 Laboratory of Parasitology and Ecology, Faculty of Science, University of Yaoundé I PO Box 812, Yaoundé-Cameroon; 3 Division of Ecology & Biodiversity, School of Biological Sciences, The University of Hong Kong, Hong Kong SAR, China; 4 Department of Ecology and Evolutionary Biology and Institute of Environment and Sustainability, University of California, Los Angeles (UCLA), Los Angeles, CA 90095, USA

**Keywords:** Climate change, Common garden, Ecotone, Thermal tolerance, *Bicyclus dorothea*

## Abstract

Thermal adaptation to habitat variability can determine species vulnerability to environmental change. For example, physiological tolerance to naturally low thermal variation in tropical forests species may alter their vulnerability to climate change impacts, compared with open habitat species. However, the extent to which habitat-specific differences in tolerance derive from within-generation versus across-generation ecological or evolutionary processes are not well characterized. Here we studied thermal tolerance limits of a Central African butterfly (*Bicyclus dorothea*) across two habitats in Cameroon: a thermally stable tropical forest and the more variable ecotone between rainforest and savanna. Second generation individuals originating from the ecotone, reared under conditions common to both populations, exhibited higher upper thermal limits (CTmax) than individuals originating from forest (∼3°C greater). Lower thermal limits (CTmin) were also slightly lower for the ecotone populations (∼1°C). Our results are suggestive of local adaptation driving habitat-specific differences in thermal tolerance (especially CTmax) that hold across generations. Such habitat-specific thermal limits may be widespread for tropical ectotherms and could affect species vulnerability to environmental change. However, microclimate and within-generation developmental processes (e.g. plasticity) will mediate these differences, and determining the fitness consequences of thermal variation for ecotone and rainforest species will require continued study of both within-generation and across-generation eco-evolutionary processes.

This article has an associated First Person interview with the first author of the paper.

## Abstract (precis)

**Summary:** The thermal tolerance of *Bicyclus dorothea* is habitat-specific with higher CTmax in ecotone populations compared to their forest counterparts, while CTmin was relatively similar across habitats.

## INTRODUCTION

It is presently widely accepted that ongoing climate warming has clear and widespread consequences for biodiversity including local extinction, population declines, shifts in community structure and composition, and changes in phenology (Scheffers et al., 2014). Physiologically based models show that tropical ectotherms may be more vulnerable to climate warming than temperate species by virtue of being adapted to lower thermal variation (Deutsch et al., 2008). However, the sensitivity of ectotherms to warming is also structured by experienced thermal variation across elevation and habitat (Sunday et al., 2014; García-Robledo et al., 2016). For species with broad distributions, populations may occur in diverse habitat types where selective pressures (e.g. temperature) during ontogeny may act differentially and have significant repercussions on physiological traits (Hoffmann et al., 2003). Local adaptation to thermal conditions can thus lead to population-specific responses (Somero, 2010; Kaspari et al., 2015; Nadeau et al., 2017).

Recent studies in tropical ecosystems have found that forest species of ectotherms tend to have lower tolerance to warming because they are restricted to more stable thermal regimes and might be especially vulnerable to climate change compared with more open habitat species (Huey et al., 2009; Frishkoff et al., 2015; Bonebrake et al., 2016; Nowakowski et al., 2017). However, ectotherms do have the ability to behaviorally avoid extreme temperatures through thermoregulatory activities and experiencing diverse microclimates at multiple spatial scales (Sunday et al., 2014; Bonebrake et al., 2014; Pincebourde and Woods, 2020). Furthermore, thermal tolerance differences in populations across habitats can sometimes be diminished by developmental conditions; Montejo-Kovacevich et al. (2020), for example, found that for tropical *Heliconius erato* butterflies, differences in heat tolerance across elevation were nearly erased after rearing in a common garden.

Measured thermal traits are ultimately a combination of natural selection/genetics, intergenerational plasticity (offspring traits affected by parental environmental conditions), developmental plasticity, and acclimation or reversible plasticity (Llewelyn et al., 2018). Indeed, acclimation within generations can drive CTmax variation for amphibians across habitats (Kristensen et al., 2008; Gunderson and Stillman, 2015; Simon et al., 2015). Upper thermal limits for terrestrial ectotherms appear to be fairly constrained – but details for how such limits vary with acclimation and plasticity across habitats or microhabitats are needed (Hoffmann et al., 2013). Specifically, knowing the extent to which differences in habitat-specific thermal tolerance can be explained by processes within or across generations can help elucidate vulnerability to climate change, especially for tropical ectotherms.

Though tropical Africa is dominated by rainforest, there is important variation in ecosystem physiognomy; ecotones that consist of a mosaic of woody or herbaceous savanna and gallery forests representing a key habitat in the landscape and region thought to be important for speciation (Smith et al., 1997). Rainforest climates tend to be constant and regular due to high tree density and high annual average precipitation. Ecotones in contrast have relatively variable climates and consist of a mosaic of homogenous vegetation types leading to more open canopy spaces than rainforests. In this study we investigated the role of habitat in structuring thermal limits, critical thermal maximum (CTmax) and minimum (CTmin), for *Bicyclus dorothea* (Cramer, 1779), a nymphalid butterfly found in both forest and ecotone habitats in many tropical African countries (Dongmo et al., 2017). *Bicyclus dorothea* exhibits differential variability in wing morphology across ecotone and forest habitats ecotone in Cameroon (Dongmo et al., 2018). We hypothesized that *B. dorothea* populations from the ecotone would have wider thermal tolerance breadths than forest populations (reflecting the greater thermal variation in those environments) that would be preserved after lab-rearing under common conditions.

## RESULTS

We collected CTmin and CTmax data for a total of 399 s generation butterflies originating across all sites. The mean CTmin (±s.e.) for ecotone females (4.5±0.15, *n=110*) and males (4.4±0.14, *n=106*) was lower than forest females (5.1±0.16, *n*=89) and males (4.9±0.15, *n*=94); habitat affected CTmin (*P<*0.001) while sex and site did not (*P>*0.50; Fig. 2, Table 1). The mean CTmax (±s.e.) for ecotone females (45.9±0.14, *n*=110) and males (46.1±0.15, *n*=106) was higher than forest females (43.27±0.18, *n*=89) and males (43.9±0.13, *n*=94). CTmax was statistically different between habitat (*P<*0.001) and sampling site (*P<*0.001) and also for sex (*P*=0.01; Fig. 2, Table 1). The results for thermal tolerance breadth were qualitatively the same as those for CTmax (Table 1).

**Table 1.**
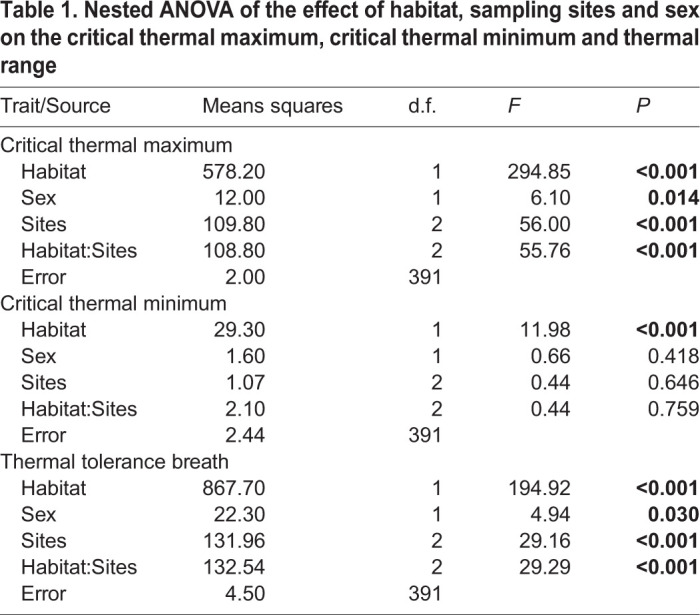
**Nested ANOVA of the effect of habitat, sampling sites and sex on the critical thermal maximum, critical thermal minimum and thermal range**

Simulated microclimatic variation demonstrated that ecotone sites exhibit lower minimum and higher maximum temperatures than forest sites (Fig. 3). Shade and height have little influence on minimum temperatures, but 1 cm maximum temperatures with 0% shade are considerably higher (by over 10°C) than both 120 cm and 1 cm with 100% shade maximum temperatures (which are very similar to one another; Fig. 3). Relating to the measured thermal tolerance limits, mean CTmin estimates were much lower than simulated minimum temperatures (about 10°C lower) while mean CTmax estimates were comparable to simulated maximum temperatures at 1 cm with 0% shade (Fig. 3).

## DISCUSSION

**Fig. 1.**
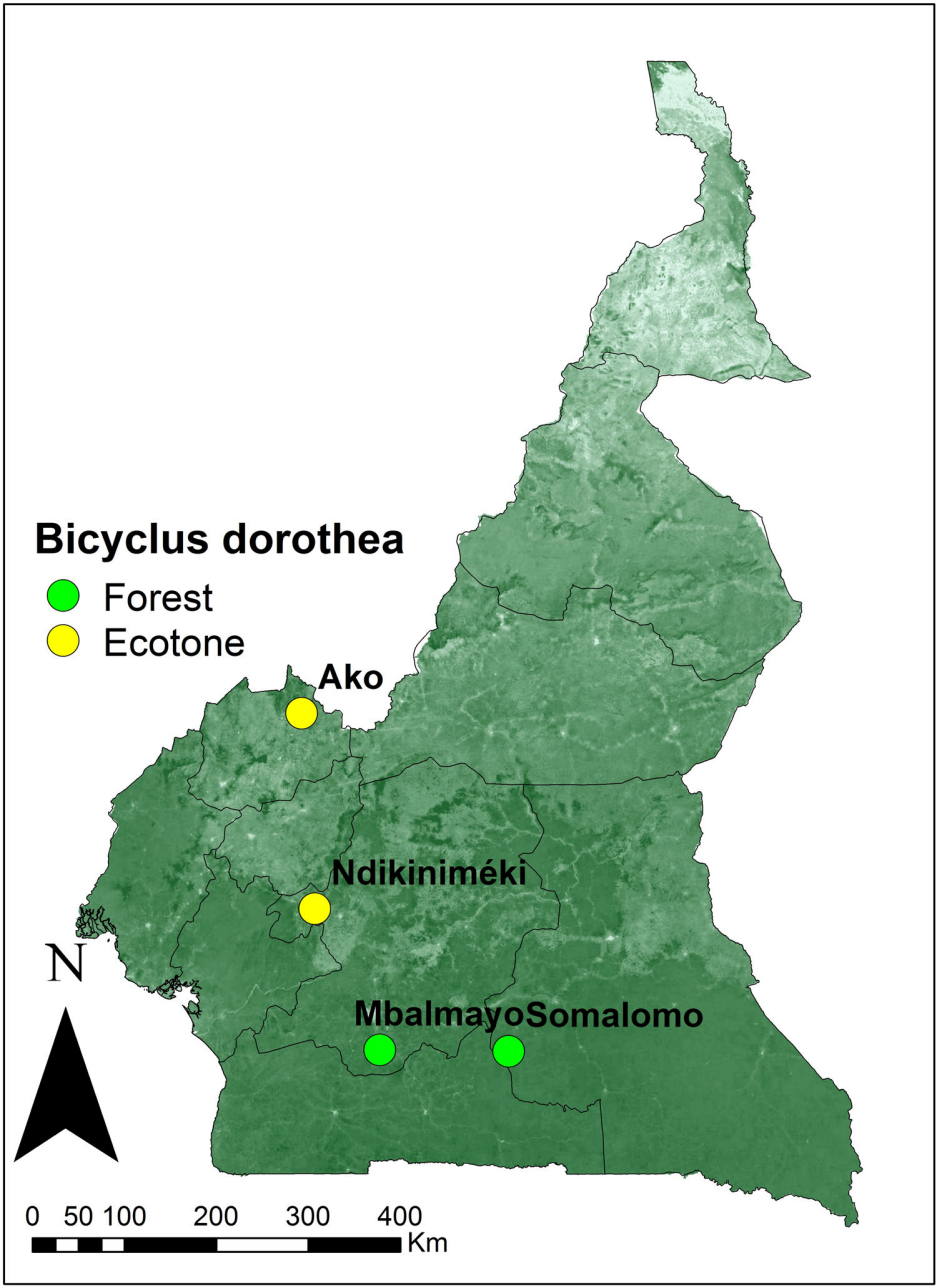
**Sampling localities of *B. dorothea* across different habitats in Cameroon.** Base map represents forest cover as estimated from land cover GLC2000 (http://www.diva-gis.org/gdata).

The higher CTmax (by ∼3°C) in ecotone *B. dorothea* populations relative to forest populations, assessed using second generation individuals reared in a common environment, indicates a level of habitat-specific adaptation in thermal tolerance for this species. We also found that CTmin was lower for ecotone populations, but to a smaller extent (by ∼1°C). At least for large spatial gradients then (hundreds of kilometers, Fig. 1), our results show that local adaptation to habitat climatic conditions may result in differential thermal tolerances for species which may have consequences for vulnerability to environmental and climatic change.

**Fig. 2.**
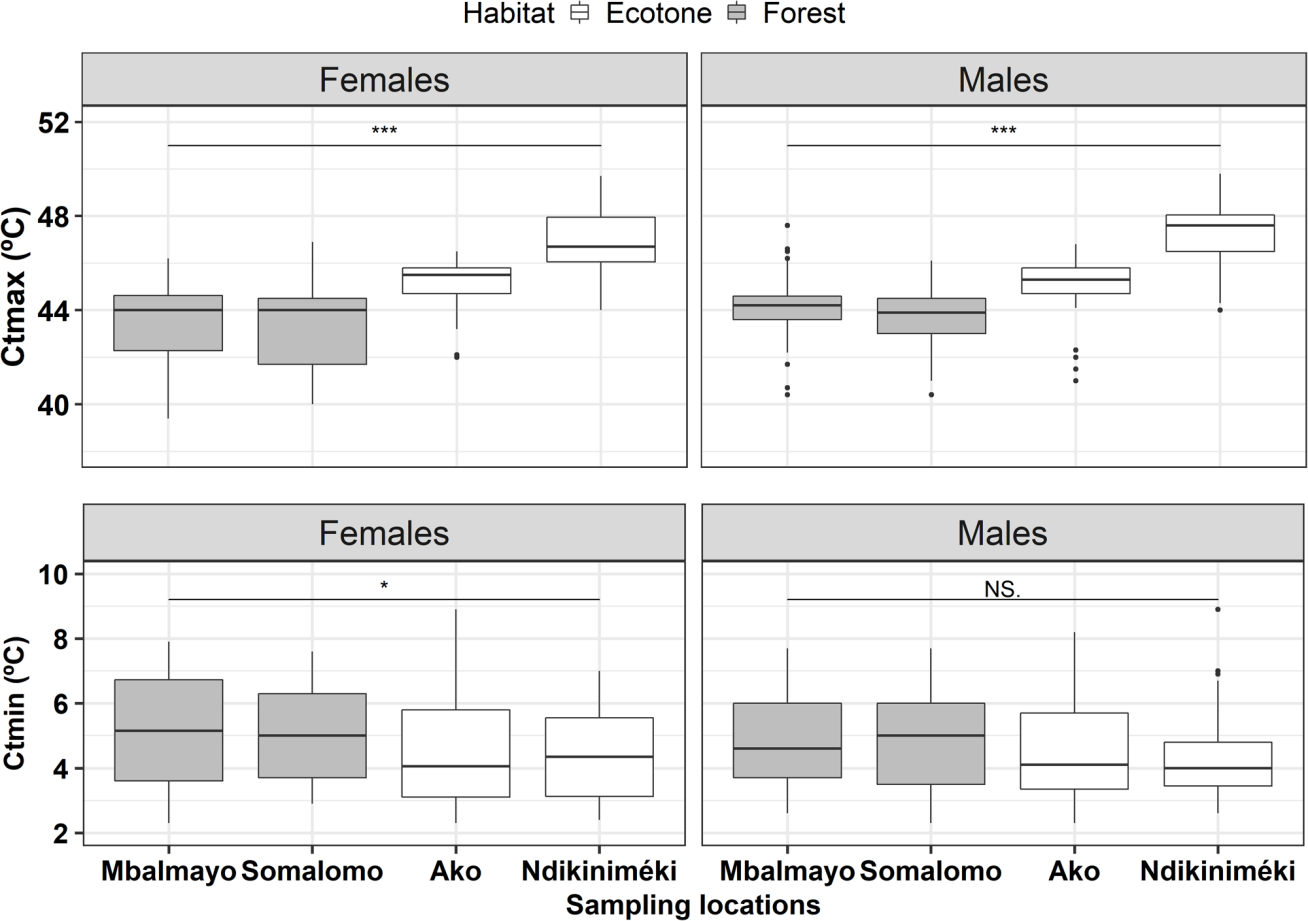
**Critical thermal maximum (A) and minimum (B) for the second-generation individuals originating from four different populations of *B. dorothea* belonging to two contrasted habitats (forest versus ecotone) in Cameroon.** Significant effects are shown via *t*-tests between habitat for males and females; for each sampling location, dots represent the outliers (for CTmin and CTmax), the boxes represent the distribution of the 50% of the values obtained for each trait (CTmin and CTmax). *P*-values significance: ***P<0.001, *P=0.008, NS: non significant *P*=0.714.

Our results support the evidence of local adaptation playing a role in variable thermal tolerance limits across habitat, as has been shown in a variety of systems including tropical beetles by García-Robledo et al. (2016) and tropical lizards by Moritz et al. (2012). However, variation in thermal limits can also be a consequence of developmental effects, ontogenetic changes, and phenotypic plasticity (Bowler and Terblanche, 2008; Kellermann and Sgrò, 2018). As an example, and in contrast with our own findings, Montejo-Kovacevich et al. (2020) found little difference in thermal tolerance (knockdown time) for a tropical butterfly species across elevation following rearing in a common garden environment, despite finding significant differences in wild-caught individuals from high versus low elevation populations.

**Fig. 3.**
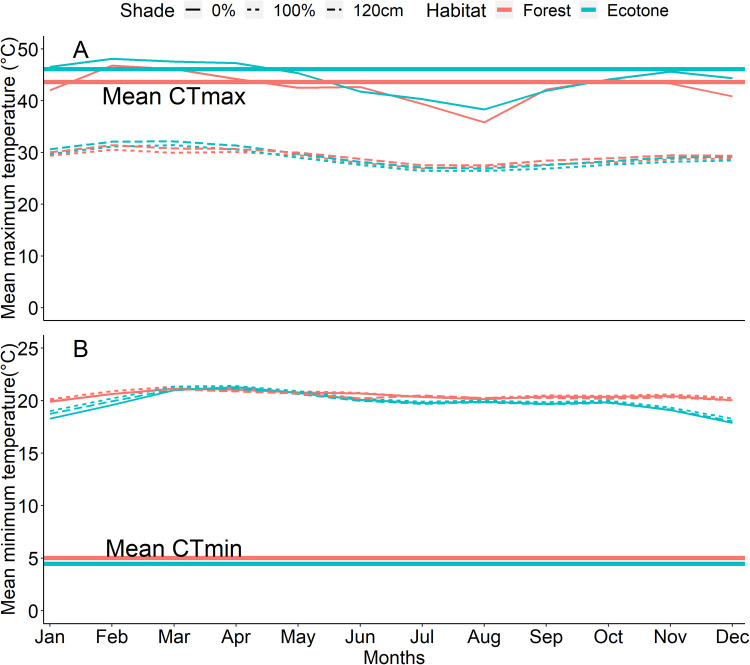
**Simulated mean maximum (A) and minimum (B) temperatures under variable conditions (1 cm 0% shade, 1 cm 100% shade, and 120 cm) across habitats using the microclim dataset (Kearney et al., 2014).** Mean CTmax (A) and CTmin (B) for forest versus ecotone populations represented by horizontal lines, assessed using second-generation individuals reared under common garden conditions in the laboratory.

Following the terminology of Hoffmann and Sgro (2018), we have, through our experimental set up, been able to infer a genetic (across-generation) basis of thermal traits in *B. dorothea* across habitats, which may be an indication of local adaptation; however, we have not been able to determine what the field fitness consequences of these differences might be. Through simulations of microclimatic variation across habitat (Fig. 3), there is evidence that the CTmax differences between ecotone and forest are very similar to the maximum temperature differences at 1 cm with no shade. The observed CTmax differences therefore may have some ecological relevance to the experienced thermal variation. However, the microclimatic data also demonstrated how temperatures higher off the ground (120 cm) and in the shade lower maximum temperatures considerably (Fig. 3). These are conditions under which *B. dorothea* are commonly active. This result again emphasizes the importance of microclimates, and solar radiation in particular, in determining vulnerability or resilience of small ectotherms to extreme temperatures (Bonebrake et al., 2014; Pincebourde and Suppo, 2016). CTmin was much lower than modeled minimum temperatures, which may explain why the observed differences across habitats were minimal. This result also suggests that CTmin may not have great consequences for *B. dorothea* for our study sites.

We know from previous study of *B. dorothea* in Cameroon in the field, that habitat-specific differences in other traits (wing phenotypes) are clear between ecotone and forest (Dongmo et al., 2018). This then further supports the evidence for local adaptation within habitats. However, to date, no study has explored dispersal capacity, though the species does not appear to fly long distances regularly (Dongmo et al., 2017). In-depth genetic study would be ideal for better revealing the underlying processes structuring thermal tolerance and other trait differences across habitats. Research across the same ecotone-forest gradient in Cameroon, has shown evidence of morphological and genetic differentiation in the lizard *Trachylepis affinis* (Freedman et al., 2010) in addition to thermal performance variation similar to our findings, i.e. ∼3°C higher thermal optimum and ∼1°C higher CTmax for wild-caught ecotone lizards (Landry Yuan et al., 2018).

Other than the lack of field fitness study in our system, other limitations of this study are important for interpretation of our results. While we found different thermal limits across habitats for populations reared under a shared environment, all colonies were reared at a single temperature (26°C). The magnitude of responses of all populations to heat and cold stresses may have been less differentiated if populations were acclimated at different temperatures. We also used a fast-ramping rate (0.5°C per minute), which may increase the relevance of our results to warming (Rezende et al., 2011), but the precise ecological relevance of this ramping rate in our system remains unknown. Furthermore, heat hardening (short-term acclimation to warm temperatures) has been demonstrated in *Bicyclus anynana* (Fischer et al., 2010) such that short-term plastic responses to thermal variation may be key in *B. dorothea* and other tropical insects. Ultimately, insect responses to climate change will be the result of a complex interplay between behavior, phenology, evolution and plasticity in response to thermal variation across multiple temporal scales (Bonebrake et al., 2014; Kingsolver and Buckley, 2017).

## MATERIAL AND METHODS

### Sampling sites

*Bicyclus dorothea* sampling took place in four localities in Cameroon (Fig. 1). Two sites represented tropical rainforest: one in a forest near the locality of Mbalmayo (N 3.388, E 11.47, alt. 768 m above sea level) characterized by a degraded secondary forest, and another site in Somalomo (N 3.37405, E 12.7332, alt. 638 m above sea level) with a mature forest located in the Dja faunal reserve. Two other sites represented ecotone habitats: Ako (N 6.68783, E 10.70687, alt. 706 m) and Ndikiniméki (N 4.76986, E 12.7332, alt. 812 m), both characterized by rainforest-savanna mosaic (Fig. 1).

For each site we modelled local microclimatic variation using microclim simulations (Kearney et al., 2014) at variable heights and shade conditions. At each locality (from a ∼15 km resolution), estimates of hourly minimum and maximum temperatures on a long-term climatology were downloaded (Hijmans et al., 2005; Kearney et al., 2014). Twenty-four layers (one for each hour of the day from 0:00 h to 23:00 h) of hourly temperature were downloaded from which maximum and minimum temperature values for each latitude/longitude of the study sites were extracted with the packages ‘raster’ (Hijmans, 2020) and ‘ncdf4’ (Pierce, 2019). We then took the mean monthly values for multiple microclimatic conditions: (1) 1 cm from the ground with 0% shade, (2) 1 cm from the ground with 100% shade, and (3) 120 cm from the ground (Kearney et al., 2014). As *B. dorothea* is an understory species typically flying low to the ground under variable levels of canopy cover, then these conditions are likely to be well representative of the microclimatic variation experienced by individuals in the sites.

### Establishment of *B. dorothea* laboratory colonies

Laboratory colonies from each site were established with adult female butterflies collected with overripe-banana baited traps and hand-net captures. Sampling of wild adult butterflies was conducted during the wet season, which runs from April to October while the dry season generally starts in early November and ends in March (for all sites). The wet season was chosen because *B. dorothea* adults are scarce and generally cease reproduction during the dry season (Dongmo et al., 2017), while during the wet season, resources are abundant, matings are common, and females can lay large quantities of fertile eggs. For logistical reasons, sampling was then carried out in July 2016 for the localities of Ako and Ndikiniméki and in May 2017 for Mbalmayo and Somalomo.

Adult butterflies collected at each locality were brought to the laboratory at the International Institute of Tropical Agriculture (IITA) in Yaoundé, Cameroon. For each population, at least 50 gravid females were captured from the field and kept in cages (ten individuals per cage) with mashed banana, distilled water and potted millet [*Pennisetum glaucum* (L.) R. Br., 1810] was used as the egg-laying host plant. Eggs (F1) laid by the field-collected females were collected and kept in Petri dishes lined with a moistened filter paper until hatching. Hatchlings (from all populations) were reared in population cages (24×24×24 cm, made of polyester screen to facilitate air circulation) and fed on potted lawn [*Axonopus compressus* (Sw.) P. Beauv., 1812] in a room where temperature was maintained at a constant 26°C and high relative humidity at 80%, and a 12D:12L photoperiod. Pupae were collected daily and transferred to individual cages where they developed to eclosion. Under these room conditions the mean development time (from egg to adult) was about 45 days.

To avoid maternal effects on thermal tolerance, all adult butterflies used in this experiment were from the second generation (F2) of the laboratory colony. Second generation butterflies of each population were obtained by allowing first generation adults to mate randomly with other adults who also originated from the same initial population cage exposed in a room where the conditions were the same as in the rearing of the first-generation individuals, i.e. 26°C and 80% relative humidity and a photoperiod of 12D:12L. Each of these cages (24×24×24 cm) contained ten adults (five males and five females) from the first generation, mashed ripe banana, cotton soaked with distilled water and potted millet *P. glaucum* as the egg-laying host plant, which was changed as needed. Multiple cohorts of butterflies (from all four sites) were reared between August 2016 and November 2017.

### Thermal tolerance

To measure critical thermal minimum (CTmin), 1-day-old second-generation adult butterflies belonging to each of the population cages (four in total for each locality) were placed individually in small plastic 250 ml cups with about 25 holes (about 5 mm diameter to allow good air circulation with the environmental chamber) in their wall and were placed in an environmental chamber (I-36VL Percival Scientific Inc., Perry, IA, USA) initially set at 26°C (Fischer et al., 2010). After 1 h, the climate cabinet was set to a ramping mode with temperature decreasing at rate of 0.5°C per minute (Piyaphongkul et al., 2012). We observed butterflies’ reaction to temperature variation through a window incorporated into the main door of the climate cabinet (which remained closed during the procedure). To monitor the real-time temperature inside the climate cabinet, a water proof thermometer probe (DE:30W, DER EE, New Taipei City, Taiwan) was displayed inside the climate cabinet in such a way that values on the thermometer could be read easily through the window of the climate cabinet. The critical thermal minimum was the coldest temperature at which adult butterflies were not able to flap their wings or make any movement with their appendages.

For CTmax, 1-day-old butterflies were also used. The same method was used but the climate cabinet was set in a ramping mode with increasing temperature at the same rate (0.5°C per minute). The critical thermal maximum was the high temperature at which each butterfly was not able stand on their legs (Huey and Stevenson, 1979; Lutterschmidt and Hutchison, 1997). For each individual butterfly, we first measured the critical thermal minimum and after that, it was taken back from the climate cabinet to the room maintained at 26°C for recovery. The critical thermal maximum was assessed on each recovered individual 24 h later. Individuals that died after the assessment of the critical thermal minimum were excluded from the analysis. From the CTmin and CTmax values of each individual, we calculated the thermal tolerance breath of each individual as the difference between CTmax and CTmin.

### Data analysis

The effects of habitat, sex and sampling site on the critical thermal minimum, maximum, and tolerance breadth were analyzed using a nested ANOVA model, with sampling sites nested in habitat (forest and ecotone). In order to meet ANOVA requirements, data were log-transformed. Pair-wise comparisons were performed with Tukey's HSD. All statistical analyses were done in R version 3.5.1 (R core team, 2019). Thermal tolerance data is available through the Data Dryad repository.

### Ethical statement

All research was carried out in accordance with permission by the Ministry of Scientific Research and Innovation of Cameroon (MINRESI).

## References

[BIO058619C1] Bonebrake, T. C., Boggs, C. L., Stamberger, J. A., Deutsch, C. A. and Ehrlich, P. R. (2014). From global change to a butterfly flapping: biophysics and behaviour affect tropical climate change impacts. *Proc. R. Soc. B Biol. Sci.* 281, 20141264. 10.1098/rspb.2014.1264 PMC4173678 25165769

[BIO058619C2] Bonebrake, T. C., Pickett, E. J., Tsang, T. P. N., Tak, C. Y., Vu, M. Q. and Vu, L. V. (2016). Warming threat compounds habitat degradation impacts on a tropical butterfly community in Vietnam. *Glob. Ecol. Conserv.* 8, 203-211. 10.1016/j.gecco.2016.09.003

[BIO058619C3] Bowler, K. and Terblanche, J. S. (2008). Insect thermal tolerance: what is the role of ontogeny, ageing and senescence? *Biol. Rev.* 83, 339-355. 10.1111/j.1469-185X.2008.00046.x 18979595

[BIO058619C4] Deutsch, C. A., Tewksbury, J. J., Huey, R. B., Sheldon, K. S., Ghalambor, C. K., Haak, D. C. and Martin, P. R. (2008). Impacts of climate warming on terrestrial ectotherms across latitude. *Proc. Natl. Acad. Sci. USA* 105, 6668-6672. 10.1073/pnas.0709472105 18458348 PMC2373333

[BIO058619C5] Dongmo, M. A. K., Bonebrake, T. C., Fomena, A. and Hanna, R. (2017). Life history notes on *Bicyclus dorothea* Cramer (Nymphalidae: Satyrinae) in Cameroon. *Trop. Lepidop. Res.* 27, 28-32.

[BIO058619C6] Dongmo, M. A. K., Bonebrake, T. C., Hanna, R. and Fomena, A. (2018). Seasonal polyphenism in *Bicyclus dorothea* (Lepidoptera: Nymphalidae) across different habitats in Cameroon. *Environ. Entomol.* 47, 1601-1608. 10.1093/ee/nvy135 30219832

[BIO058619C7] Fischer, K., Dierks, A., Franke, K., Geister, T. L., Liszka, M., Winter, S. and Pflicke, C. (2010). Environmental effects on temperature stress resistance in the tropical butterfly *Bicyclus Anynana*. *PLoS ONE* 5, e15284. 10.1371/journal.pone.0015284 21187968 PMC3004918

[BIO058619C8] Freedman, A. H., Thomassen, H. A., Buermann, W. and Smith, T. B. (2010). Genomic signals of diversification along ecological gradients in a tropical lizard. *Mol. Ecol.* 19, 3773-3788. 10.1111/j.1365-294X.2010.04684.x 20618893

[BIO058619C9] Frishkoff, L. O., Hadly, E. A. and Daily, G. C. (2015). Thermal niche predicts tolerance to habitat conversion in tropical amphibians and reptiles. *Glob. Change Biol.* 21, 3901-3916. 10.1111/gcb.13016 26148337

[BIO058619C10] García-Robledo, C., Kuprewicz, E. K., Staines, C. L., Erwin, T. L. and Kress, W. J. (2016). Limited tolerance by insects to high temperatures across tropical elevational gradients and the implications of global warming for extinction. *Proc. Natl. Acad. Sci. USA* 113, 680-685. 10.1073/pnas.1507681113 26729867 PMC4725502

[BIO058619C11] Gunderson, A. R. and Stillman, J. H. (2015). Plasticity in thermal tolerance has limited potential to buffer ectotherms from global warming. *Proc. R. Soc. B Biol. Sci.* 282, 20150401. 10.1098/rspb.2015.0401 PMC4455808 25994676

[BIO058619C12] Hijmans. (2020). Raster: Geographic Data Analysis and Modeling. R package version 3.3-13. https://CRAN.R-project.org/package=raster.

[BIO058619C13] Hijmans, R. J., Cameron, S. E., Parra, J. L., Jones, P. G. and Jarvis, A. (2005). Very high resolution interpolated climate surfaces for global land areas. *Int. J. Climatol.* 25, 1965-1978. 10.1002/joc.1276

[BIO058619C14] Hoffmann, A. A. and Sgrò, C. M. (2018). Comparative studies of critical physiological limits and vulnerability to environmental extremes in small ectotherms: how much environmental control is needed? *Int. Zool.* 13, 355-371. 10.1111/1749-4877.12297 PMC6099205 29168624

[BIO058619C15] Hoffmann, A. A., Sørensen, J. G. and Loeschcke, V. (2003). Adaptation of *Drosophila* to temperature extremes: bringing together quantitative and molecular approaches. *J. Therm. Biol* 28, 175-216. 10.1016/S0306-4565(02)00057-8

[BIO058619C16] Hoffmann, A. A., Chown, S. L. and Clusella-Trullas, S. (2013). Upper thermal limits in terrestrial ectotherms: how constrained are they? *Funct. Ecol.* 27, 934-949. 10.1111/j.1365-2435.2012.02036.x

[BIO058619C17] Huey, R. B. and Stevenson, R. D. (1979). Integrating thermal physiology and ecology of ectotherms: a discussion of approaches. *Am. Zool.* 19, 357-366. 10.1093/icb/19.1.357

[BIO058619C18] Huey, R. B., Deutsch, C. A., Tewksbury, J. J., Vitt, L. J., Hertz, P. E., Álvarez Pérez, H. J. and Garland, T. (2009). Why tropical forest lizards are vulnerable to climate warming. *Proc. R. Soc. B Biol. Sci.* 276, 1939-1948. 10.1098/rspb.2008.1957 PMC2677251 19324762

[BIO058619C19] Kaspari, M., Clay, N. A., Lucas, J., Yanoviak, S. P. and Kay, A. (2015). Thermal adaptation generates a diversity of thermal limits in a rainforest ant community. *Glob. Change Biol.* 21, 1092-1102. 10.1111/gcb.12750 25242246

[BIO058619C20] Kearney, M. R., Isaac, A. P. and Porter, W. P. (2014). microclim: global estimates of hourly microclimate based on long-term monthly climate averages. *Sci. Data* 1, 140006. 10.1038/sdata.2014.6 25977764 PMC4387738

[BIO058619C21] Kellermann, V. and Sgrò, C. M. (2018). Evidence for lower plasticity in CT_max_ at warmer developmental temperatures. *J. Evol. Biol.* 31, 1300-1312. 10.1111/jeb.13303 29876997

[BIO058619C22] Kingsolver, J. G. and Buckley, L. B. (2017). Evolution of plasticity and adaptive responses to climate change along climate gradients. *Proc. R. Soc. B Biol. Sci.* 284, 20170386. 10.1098/rspb.2017.0386 PMC5563792 28814652

[BIO058619C23] Kristensen, T. N., Hoffmann, A. A., Overgaard, J., Sørensen, J. G., Hallas, R. and Loeschcke, V. (2008). Costs and benefits of cold acclimation in field-released *Drosophila*. *Proc. Natl. Acad. Sci. USA* 105, 216-221. 10.1073/pnas.0708074105 18162547 PMC2224189

[BIO058619C24] Landry Yuan, F., Freedman, A. H., Chirio, L., LeBreton, M. and Bonebrake, T. C. (2018). Ecophysiological variation across a forest-ecotone gradient produces divergent climate change vulnerability within species. *Ecography* 41, 1627-1637. 10.1111/ecog.03427

[BIO058619C25] Llewelyn, J., Macdonald, S. L., Moritz, C., Martins, F., Hatcher, A. and Phillips, B. L. (2018). Adjusting to climate: acclimation, adaptation and developmental plasticity in physiological traits of a tropical rainforest lizard. *Integr. Zool.* 13, 411-427. 10.1111/1749-4877.12309 29316349

[BIO058619C26] Lutterschmidt, W. I. and Hutchison, V. H. (1997). The critical thermal maximum: data to support the onset of spasms as the definitive end point. *Can. J. of Zool.* 75, 1553-1560. 10.1139/z97-782

[BIO058619C27] Montejo-Kovacevich, G., Martin, S. H., Meier, J. I., Bacquet, C. N., Monllor, M., Jiggins, C. D. and Nadeau, N. J. (2020). Microclimate buffering and thermal tolerance across elevations in a tropical butterfly. *J. Exp. Biol.* 223, jeb220426. 10.1242/jeb.220426 32165433 PMC7174841

[BIO058619C28] Moritz, C., Langham, G., Kearney, M., Krockenberger, A., VanDerWal, J. and Williams, S. (2012). Integrating phylogeography and physiology reveals divergence of thermal traits between central and peripheral lineages of tropical rainforest lizards. *Philos. Trans. R. Soc. B Biol. Sci.* 367, 1680-1687. 10.1098/rstb.2012.0018 PMC3350659 22566675

[BIO058619C29] Nadeau, C. P., Urban, M. C. and Bridle, J. R. (2017). Climates past, present, and yet-to-come shape climate change vulnerabilities. *Trends Ecol. Evol.* 32, 786-800. 10.1016/j.tree.2017.07.012 28844791

[BIO058619C30] Nowakowski, A. J., Watling, J. I., Whitfield, S. M., Todd, B. D., Kurz, D. J. and Donnelly, M. A. (2017). Tropical amphibians in shifting thermal landscapes under land-use and climate change. *Conserv. Biol.* 31, 96-105. 10.1111/cobi.12769 27254115

[BIO058619C31] Pierce. (2019). ncdf4: Interface to Unidata netCDF (Version 4 or Earlier) Format Data Files. R package version 1.17. https://CRAN.R-project.org/package=ncdf4.

[BIO058619C32] Pincebourde, S. and Suppo, C. (2016). The vulnerability of tropical ectotherms to warming is modulated by the microclimatic heterogeneity. *Integr. Comp. Biol.* 56, 85-97. 10.1093/icb/icw014 27371561

[BIO058619C33] Pincebourde, S. and Woods, H. A. (2020). There is plenty of room at the bottom: microclimates drive insect vulnerability to climate change. *Curr. Opin. Insect Sci.* 41, 63-70. 10.1016/j.cois.2020.07.001 32777713

[BIO058619C34] Piyaphongkul, J., Pritchard, J. and Bale, J. (2012). Can tropical insects stand the heat? A case study with the brown planthopper *Nilaparvata lugens* (Stål). *PLoS ONE* 7, e29409. 10.1371/journal.pone.0029409 22253720 PMC3257224

[BIO058619C35] Rezende, E. L., Tejedo, M. and Santos, M. (2011). Estimating the adaptive potential of critical thermal limits: methodological problems and evolutionary implications. *Funct. Ecol.* 25, 111-121. 10.1111/j.1365-2435.2010.01778.x

[BIO058619C36] Scheffers, B. R., Edwards, D. P., Diesmos, A., Williams, S. E. and Evans, T. A. (2014). Microhabitats reduce animal's exposure to climate extremes. *Glob. Change Biol.* 20, 495-503. 10.1111/gcb.12439 24132984

[BIO058619C37] Simon, M. N., Ribeiro, P. L. and Navas, C. A. (2015). Upper thermal tolerance plasticity in tropical amphibian species from contrasting habitats: Implications for warming impact prediction. *J. Therm. Biol.* 48, 36-44. 10.1016/j.jtherbio.2014.12.008 25660628

[BIO058619C38] Smith, T. B., Wayne, R. K., Girman, D. J. and Bruford, M. W. (1997). A role for ecotones in generating rainforest biodiversity. *Science* 276, 1855-1857. 10.1126/science.276.5320.1855

[BIO058619C39] Somero, G. N. (2010). The physiology of climate change: how potentials for acclimatization and genetic adaptation will determine 'winners’ and 'losers’. *J. Exp. Biol.* 213, 912-920. 10.1242/jeb.037473 20190116

[BIO058619C40] Sunday, J. M., Bates, A. E., Kearney, M. R., Colwell, R. K., Dulvy, N. K., Longino, J. T. and Huey, R. B. (2014). Thermal-safety margins and the necessity of thermoregulatory behavior across latitude and elevation. *Proc. Natl. Acad. Sci. USA* 111, 5610-5615. 10.1073/pnas.1316145111 24616528 PMC3992687

[BIO058619C41] R Core Team. (2019). *R: A Language and Environment for Statistical Computing*. Vienna, Austria: R Found. Stat. Comput. www.R-project.org.

